# Factors Influencing Sense of Coherence: Family Relationships, High School Life and Autism Spectrum Tendency

**DOI:** 10.3390/children7090108

**Published:** 2020-08-21

**Authors:** Tomoko Omiya, Naoko Deguchi, Taisuke Togari, Yoshihiko Yamazaki

**Affiliations:** 1 Public Health Nursing, Faculty of Medicine, University of Tsukuba, Tsukuba 305-8575, Japan; naokok-tky@umin.ac.jp; 2 Faculty of Liberal Arts, the Open University of Japan, Chiba 261-8586, Japan; ttogari@ouj.ac.jp; 3 Department of Social Welfare, Faculty of Social Welfare, Nihon Fukushi University, Chita-Gun 470-3295, Japan; yamazaki_1112@yahoo.co.jp

**Keywords:** adolescent, autism, familial relationship, school life, sense of coherence

## Abstract

Adolescence is marked by significant life stress. Recently, school refusal and dropouts as well as suicide among Japanese adolescents have increased. Sense of coherence (SOC) is recognized as a competency that helps people deal with stress. The purpose of this study was to examine the factors influencing SOC in male and female high school students. We conducted a survey with 203 pairs of high school students and their mothers, in Tokyo, to explore their SOC, family relationships, school belonging, and autistic traits. Analysis of the data revealed a weak relationship between female students’ SOC and that of their mothers, and no relationship between male students’ SOC and their mothers’ SOC. Feelings of acceptance and recognition from teachers improved students’ SOC, irrespective of gender. Low SOC in mothers had a negative impact on female students’ SOC, and children’s lack of imagination (an autism spectrum tendency) had a negative impact on male students’ SOC. This study revealed the importance of support at home and school according to the needs of both genders.

## 1. Introduction

In the early 20th century, American psychologist G.S. Hall described late adolescence as a period of “storm and stress” [1]. It is a period of psychological, social, and physiological changes; emotions tend to become unstable, and poor mental health is common [2,3]. In recent years, the number of Japanese adolescents with indefinite complaints such as general malaise, headache, and abdominal pain has been increasing, and adolescents’ mental health problems have become more severe [4]. Statistics show that a number of Japanese students are absent from school due to bullying or other interpersonal stressors [5,6]. In 2018, the number of non-attending Japanese high school students was 52,723 (1.6%) and the number of dropouts was 48,594 (1.4%); these numbers were markedly higher than the previous year (49,643 and 46,802, respectively) [7]. Moreover, for over 10 years, suicide has been the number-one cause of death among adolescents and young adults in Japan. Despite today’s declining birth rate, compared to the previous century, the number of suicides among early teens has not changed [8]. This is a particularly alarming situation from an international perspective.

In light of this background, the Ministry of Education, Culture, Sports, Science and Technology-Japan (MEXT) states that it is important to promote education that helps students learn how to deal with stress and provides an overview of common suicide prevention strategies. Young people can be at the mercy of unsolved and unprecedented problems such as globalization, changes in social conditions, and weakened family relationships. Under such stressful situations, the ability to utilize various coping resources effectively and transform a stressful experience into growth is required. MEXT has named this ability the “power to live” [9].

In recent years, sense of coherence (SOC) has gained attention as a means to cope with stress and has been the subject of extensive research, mainly in Europe, Asia, and the United States. SOC is described by Antonovsky [10] as a competency that can help one successfully cope with stress and protect mental and physical health even in extremely stressful situations, leading to healthy mental growth and development. SOC consists of three subordinate concepts: (1) comprehensibility: the sense that one can understand and explain what happened and what will happen; (2) manageability: the sense that one can deal with any problem; and (3) meaningfulness: the solution to the problems one faces, which makes one feel that it is worth the effort and the challenge. In a previous study, a survey of high school students found that a supportive school environment and SOC were related to youth health and that SOC had a direct positive effect on health [11]. In another study of adolescents, SOC was cited as one of the psychosocial factors governing health-related habits, and SOC functioned as an intermediary to maintaining a healthy lifestyle [12].

Adolescent SOC is very fragile; it is developed by the experience of building trust and having affectionate relationships [10]. In other words, a strong SOC is based on strong family ties, mutual dependence between family members, and establishment of identity [13]. Previous studies have shown the link between SOC and family and occupational environments [14]. Antonovsky [13] does not clearly state whether SOC is inherited within families; however, he notes that if the mother’s SOC is high, it is easier to provide good life experiences for the development of the child’s SOC. For example, parents with a high SOC may have a positive impact on their children’s SOC because they can provide a psychologically and socially stable family life. Understanding the extent to which SOC is affected by parents is important in elucidating the SOC formation process of a child. Few studies, except for Togari et al.’s [15], have examined the interrelationship between high school students’ SOC and that of their family members.

Adolescents spend most of their day at school; thus, much of their life experiences take place there. Feldt [16] stated that life experience at schools in Finland, especially the experience of success, was important in SOC formation. The experiences of being understood, accepted, and valued by teachers and friends, as well as successfully meeting challenges at school lead to increased self-esteem, which strengthens SOC. Togari et al. [15] showed that a high sense of belonging at school was associated with high SOC, while alienation at school was associated with low SOC. Establishing trust with friends and teachers, finding one’s own place in school, and adapting to the school environment are likely to affect SOC formation and development, but research on this is still limited.

Studies have reported that undiscovered and undiagnosed Autism Spectrum Disorder (ASD) is often behind school refusal and suicide [17,18]. Few people in Japan have been diagnosed with ASD, and many ASD adults have depression and adjustment disorders as secondary disorders [19]. It is also common for schoolteachers to think that unless students have been diagnosed with ASD, they do not need any special treatment; few parents actually submit a medical certificate asking for such consideration. ASD characteristics such as not knowing implicit rules and indulging in out-of-place behavior may lead to students’ isolation and bullying. SOC is a predictor of depression and adjustment disorders [20,21], and there may be a significant association between ASD tendencies and SOC; in fact, mothers or parents of children with ASD have low SOC [22]. However, to the best of our knowledge, no studies have examined SOC in people with ASD tendencies themselves. Although ASD has various phenotypes, it is essential to clarify what characteristics of ASD are associated with SOC in order to design appropriate interventions.

The purpose of this study was to understand whether student/mother SOC, school environment, family relationships, and ASD personality traits are related to students’ SOC.

## 2. Materials and Methods

### 2.1. Sample and Data Collection

In 2016, we provided a self-administered questionnaire to 671 students attending a Tokyo metropolitan high school and their mothers. The questionnaire was assigned a common ID number for parents and children so that they could be matched. Students’ responses were collected in a school collection box, and mothers’ responses were collected by mail. There were 629 responses from students (response rate = 93.7%) and 203 responses from mothers (response rate = 30.2%). Our analysis was conducted on the 203 pairs of mothers and students. This study focused on mothers specifically because in Japan, mothers are the main caregivers for children [23]. As inclusion criteria for parents who responded, it was clearly stated in the questionnaire that they were mothers and were the primary care providers for their children.

Takakura et al. [24] stated that in Japan, the factors associated with adolescent depression differed by gender: female students were more affected by interpersonal issues than male students. In previous longitudinal studies in Canada, the United Kingdom, and the U.S., family, school, peers, and behavior, among other factors, have been separately examined for males and females [25,26]. Based on previous studies, we decided to consider gender when studying SOC among high-school students.

### 2.2. Ethical Considerations

This study complied with the rules of the Declaration of Helsinki, revised in 2013, and was conducted with the approval of the Toho University Faculty of Nursing research ethics review committee (approval number 28,019, 27 October 2016). Since high school students formed the target group, we provided written explanations to each parent about their children’s participation in the survey, in accordance with the Japanese Ministry of Health, Labor, and Welfare ethical guidelines. We explained in the document that participation in the survey was voluntary and those who refused to take part would not be penalized. We guaranteed in writing that they could opt out of the study at any point using email, telephone, fax, letter, interview, or other means. There were no refusals from parents. We included a check box in both the parent and child questionnaires for informed consent to participate and analyzed the responses of those parents and their children who provided consent. A common number was assigned to the parent and child; the numbers did not match the student name or student ID number, and it was explained in writing that individuals could not be identified. All methods were carried out in accordance with the relevant rules, guidelines, and regulations.

### 2.3. Variables and Questionnaires

Students’ gender and year of study were recorded. The students’ mothers were asked to provide their own age, educational background, financial status, occupation, and marital status.

#### 2.3.1. Questionnaires Used for Both Mother and Child

##### Sense of Coherence (SOC)

The original SOC scale is a 29-item instrument scored on a 7-point Likert scale [27]. In this study, we used a 5-point scale, according to the Japanese version of the original SOC-13 shortened version [28]. Its reliability and validity have been established through a nationwide survey in Japan studying the average SOC score of Japanese people by age [28]. We decided to use Togari and Yamazaki’s version of this scale because we were interested in comparing data obtained from our sample with the average Japanese population. SOC-13 has three subscales: comprehensibility (5 items), manageability (4 items), and meaningfulness (4 items), from which total scores are calculated (ranging from 13–65). The Cronbach’s alpha coefficient for SOC in our study was 0.855 for mothers and 0.776 for adolescents.

##### The Family Relationships Index (FRI)

The FRI was developed based on one of the three structures of the Family Environmental Scale developed by Moos and Moos [29]. The FRI comprises three subscales that examine the family relationship dimension and was developed to measure the quality of family relationships, including support in the family environment [30]. The three subscales are (1) cohesion: the degree to which family members are engaged and committed to the family and the extent to which they are supportive of each other; (2) expressiveness: the extent to which family members act openly and express their feelings directly; and (3) conflicts: open expression of anger and attack and conflicting interactions. The FRI can be either a comprehensive index or a subscale, according to the purpose of its use. The reliability of the Japanese version of the FRI as a comprehensive index and as subscales has been verified. A score for each subscale was calculated, ranging from 12 to 48. The Cronbach’s alpha coefficients for FRI (cohesion, expressiveness, and conflict) were 0.824, 0.703, and 0.781 for mothers and 0.815, 0.705, and 0.700 for adolescents, respectively.

#### 2.3.2. Measurements for Adolescents

##### School Membership Scale

We used the Japanese short version of the school membership scale [31], which measures psychological sense of school belonging, developed by the American psychologist Goodenow [32]. It consists of three components: acceptance by students (5 items), acceptance by teachers (4 items), and sense of belonging to school (4 items). Togari et al. [31] verified the reliability and validity of the Japanese version of the scale. A total score for each subscale was calculated, ranging from 12 to 60. The questionnaire uses a 5-point Likert scale, with higher scores implying better conditions. Cronbach’s alpha of the scale in this study was 0.844.

##### Autism Spectrum Index, Japanese Version (AQ-J-16; Shortened Version)

The Autism-Spectrum Quotient (AQ), developed by Baron-Cohen, Wheelwright, Skinner, Martin, and Clubley [33], is a 50-item, self-administered questionnaire for adults with normal intelligence used to identify autism in the general public and to screen for high-functioning pervasive developmental disorder in people with an intelligence quotient (IQ) of 70 or higher. Previous studies have pointed out the relationship between a high autism spectrum index in children and low mental health and that mothers of children with developmental disabilities tend to have low SOC [22]. The AQ-J-16 used in this study is a shortened version of the Japanese scale validated by Kurita, Koyama, and Osada [34]. Items are rated on a 4-point Likert scale. Participants receive a score of 1 or 0 on each item (1 = high autism and 0 = low autism). A score of 12 points or higher implies strong autism tendencies. There are 7 items assessing low communication ability, 4 items for poor imagination, 3 items for difficulty in switching attention, and 2 items for low social skills. Scores above 12 were from 15 out of 629 students (2.4%) and 4 out of 203 (2.0%).

### 2.4. Statistical Analysis

The matched parent/child data were analyzed. First, a simple tabulation of demographic attributes and characteristics of the participants was conducted. Since normality of the distribution was found in the SOC total score, a *t*-test was performed on the average values by gender for both mothers and children. Cronbach’s alpha was calculated for each scale. Spearman’s correlation coefficients were then computed for parents and adolescents (split by gender) between the total SOC, FRI, and the subscales and other variables. We examined the correlation coefficients for each variable in order to investigate factors related to the SOC of students. Specifically, the correlation coefficients were calculated with data from the family relation scale, sense of belonging to school scores, and AQ-J-16 scores.

SPSS AMOS was used for path analysis by gender. In constructing the structural equation model, we first referred to the model of Yamazaki and Togari [35], according to which mothers’ high SOC may be related to current, good family relations, which is the path related to the high SOC of students. For family relationships, Yamazaki et al. used the mothers’ own total FRI score. In this study, we examined the FRI score of each student as well as the mother’s own variables. Note that FRI was analyzed using scores for each subscale in order to obtain specific suggestions for support. Second, we thought that the factors related to the SOC of students were school life and student ASD personality traits in addition to family relationships. We considered that school life and ASD tendencies were not related to the mothers’ SOC and examined each subscale in a different path from the mothers’ SOC. We inputted each scale’s subscale into the model and tried every pattern with the best fit. The mothers’ age and the students’ grade level (1st to 3rd grades) were used as covariates.

We used RMSEA (Root Mean Square Error of Approximation) and CFI (Comparative Fit Index) for the model fit and examined the model with CFI set to 0.9 and RMSEA set to 0.1 or less. SPSS Statistics and AMOS 25 for Windows were used for the other analyses.

## 3. Results

Nearly 90% of the mothers indicated that they were married and 42.9% stated that they were economically poor or very poor compared to 23.6% economically wealthy or very wealthy. Of the respondents, 52.7% were females, while 35.8% were first-year students—the highest number in the first to third grades. The students’ average SOC score was 37.3 ± 6.8 and the mothers’ average SOC score was 42.7 ± 7.6 (Table 1).

Table 2 shows the total SOC score differences between each group. There was no difference in mothers’ SOC scores by children’s gender. The male students’ average SOC was 38.5 ± 6.7 and the female students’ mean SOC was 35.7 ± 6.9. Male students’ SOC scores were significantly higher than those of female students (*p* = 0.004).

Table 3 shows the correlations between SOC total scores and the subscale scores of students and mothers. No relationship was found between male students’ SOC and their mothers’. A small positive correlation was found between female students’ SOC and their mothers’ (*r* = 0.226, *p* = 0.023).

There was no significant relationship between male students’ SOC scores and their mothers’ response variables. There was a small significant negative correlation (*r* = −0.200, *p* = 0.033) between conflicts in family relationships felt by mothers and female students’ SOC scores.

Table 4 shows the correlation between the students’ SOC scores (split by gender) and the following: (1) students’ FRI, (2) students’ sense of belonging to school, and (3) students’ AQJ-16 scores.

There was a positive correlation between SOC and the school membership scale scores for both male and female students (*r* = 0.414 to 0.667, *p* < 0.001). Regarding the AQ-J-16, a significant negative correlation was observed between SOC scores for both male and female students and their reduced ability to switch attention and poor communication abilities (*r* = −0.428 to −0.345, *p* < 0.001). Male students also showed a negative correlation between their SOC scores and poor imagination skills (*r* = −0.339, *p* = 0.012).

**Figure 1:**
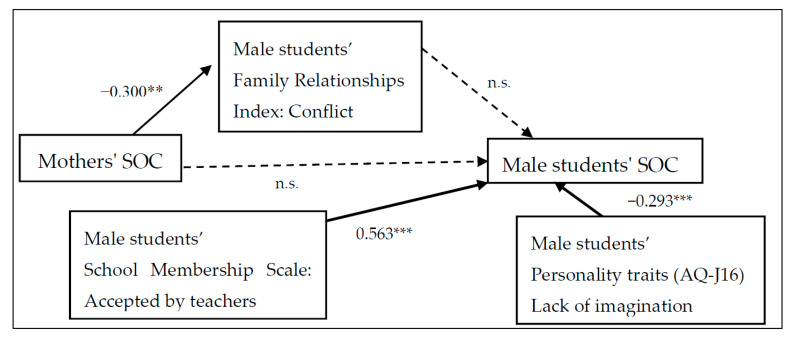
Male students’ SOC path diagram. RMSEA = 0.069, Comparative Fit Index (CFI) = 0.908, χ^2^/df = 1.95. *** *p* < 0.001, ** *p* < 0.01.

**Figure 2:**
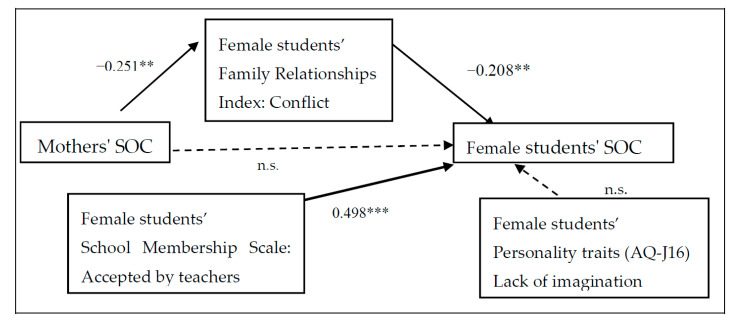
Female students’ SOC path diagram. RMSEA = 0.064, CFI = 0.908, χ^2^/df = 2.31. *** *p* < 0.001, ** *p* < 0.01.

Figure 1 and Figure 2 show the gender-specific structural equation modelling path diagrams that examine the relationship between the students’ and mothers’ SOC. The model is a good fit for the data (male: RMSEA = 0.069, CFI = 0.908; female: RMSEA = 0.064, CFI = 0.901).

For male students, the mothers’ SOC significantly affected children’s conflicting feelings about their family (*r* = −0.300, *p* < 0.010) but did not affect their SOC. Acceptance by teachers (*r* = 0.563, *p* < 0.001) had a positive impact on students’ SOC. On the other hand, lack of imagination skills as measured by the AQ-J-16 had a negative effect on students’ SOC (*r* = −0.293, *p* < 0.001). For female students, the mothers’ SOC affected children’s SOC (*r* = −0.208, *p* < 0.010) through the family conflict that the child felt (*r* = −0.351, *p* < 0.010). On the other hand, unlike the male students, the lack of imagination did not affect females’ SOC. Just as in male students, acceptance by teachers had a positive influence on the females’ SOC (*r* = 0.498, *p* < 0.001). In addition, the cohesiveness and expression of the family relationships in students and mothers, student acceptance and sense of belonging to school, AQ-J-16 communication skills, attention switching, and so forth, were examined, but a good model fit was not obtained.

## 4. Discussion

In this study, we investigated whether factors such as ASD tendencies, family relationships, and school life influenced SOC among Japanese high school students. Although SOC is said to be affected by the environment in which one grows up, so far there has been little research on the relationship between parent and child SOC. This study revealed that the factors that affect SOC were different for male and female high school students, indicating the need for gender-specific interventions to develop SOC.

### 4.1. SOC Scores

Among the participants, mothers’ SOC was higher than children’s SOC. This is in line with Antonovsky’s work indicating that SOC will rise slowly over time with age [10]. The age of mothers in our study is almost the same as the average age of the nationwide sample studied by Togari et al. (42.16 ± 8.18) [36].

Male high school students’ SOC scores were significantly higher than females’, which is consistent with previous research. A study with college students in Japan showed that males had higher SOC scores than females [37]. In a comparison of SOC in Japan, Scotland, and Canada, men showed higher SOC scores than women when young, but the difference narrowed as they got older [38,39]. The participants in this study may also exhibit similar trends.

### 4.2. SOC Relationship between Parent and Child

The female students’ total SOC scores were slightly positively correlated with their mothers’ total SOC score. Daughters are said to be closer to the mothers psychologically compared to sons [38]. In addition, particularly in Japan, mother–daughter relationships are very strong [40], which may explain this particular finding. On the other hand, for male students, there was no correlation between their SOC scores and their mothers’. Kogawa and Nishizawa [41] state that adolescent males are psychologically closer to their fathers. Thus, it will be important to investigate the correlation between male students’ and their fathers’ SOC in the future.

### 4.3. School Environment, Family Relationships, and SOC

In Togari et al.’s research targeting high school students and their mothers [15], about 88% of mothers answered that they were wealthy, compared to 23.6% in this study. All mothers were about the same age, but the mothers in the Togari et al.’s study had a higher total SOC score than those in our study. Moreover, in Togari et al.’s study, the mean SOC of male students’ mothers was 46.1, and that of female students’ mothers was 45.5, while in our study, the mean SOC score of male students’ mothers was 42.8 and that of female students’ mothers was 42.3. Regarding household wealth and SOC scores, Antonovsky and previous studies [42] found that financial wealth was associated with high SOC. Alternatively, some studies show that not experiencing financial difficulties during adolescence is associated with increased SOC [43]. From these findings, we predicted that the SOC of high school students in our study would be lower than that of the high school students Togari et al. studied. However, students’ SOC scores in both studies were about the same. This implies that high school students’ SOCs may be maintained and improved at school.

In fact, our results show that school-related variables were more strongly associated with SOC than with family relationships (Table 4). According to Antonovsky, parents who are financially rich and have high SOC can build stable family relationships, provide family management with clear rules and values, and provide a good life experience for their kids. From the results of our study, a lack of these positive experiences in the home environment can be compensated for by positive experiences at school.

Regarding the formation of SOC, putting oneself in a predictable situation fosters a sense of comprehension, and participation in decision-making and problem-solving fosters a sense of meaningfulness [35]. In high school life, rules and boundaries are clear, annual events are customary, and active participation in sports festivals and cultural festivals may be very useful for the recovery and maintenance of SOC. Further, in the path analyses (Figure 1 and Figure 2), the common finding for both males and females was that their SOC was affected by whether they felt accepted by their teachers. High school students spend much of their day at school; it seems that they feel a sense of security, permanence, and stability in their school life in accordance with the order and rules created by their teachers. In this way, school life has great potential in the SOC formation of high school students, which is a hopeful finding.

At the same time, some studies have shown that members of families with alcoholism and high levels of domestic violence and abuse also have low SOC [44]. Therefore, while a positive experience at school can offset some negative impact of the family experience, that may not happen when the family experience is severely deficient. For a greater generalization of the results, a more detailed study is required on family and school experiences, as well as the type of school (private or public school), its geographical location (rural or urban), the students’ level of academic ability, and so forth.

### 4.4. Female Students’ SOC

Adolescent females perceived that the mothers’ SOC affected family conflict, thereby impacting the daughter’s SOC. This study used structural equation modelling, but it is also possible that the daughters’ own SOC caused stress, anxiety, and domestic conflict that affected the mothers’ SOC. Previous studies have revealed that irritable temperament is negatively associated with SOC [45], and adolescence is a period of conflict and irritability [46]. Therefore, attention has been directed increasingly toward the role that children’s and adolescents’ ability to regulate their own emotions plays in the development of these difficulties [47]. In any case, a hostile home atmosphere can be especially damaging to daughters’ SOC. A longitudinal study could throw light upon whether that is due to the daughters’ low SOC or the mothers’ temperament or SOC. It may also be necessary to provide input to families on how to deal with their daughters’ anger. Some studies showed that mothers’ coaching helped soothe adolescents’ emotions of irritability and anger [47]. In order to preserve and enhance one’s SOC, it may also be important to deepen parents’ understanding of adolescent irritation.

### 4.5. Male Students’ SOC

For male students, similar to female students, our results suggested that if the mother’s SOC was low, the son felt a high degree of family conflict. However, for male students, this did not affect their own SOC. In contrast, the lack of imagination as an ASD tendency had a negative impact on male students’ SOC. Examples of items measuring lack of imagination include “It is difficult for me to understand the intentions of the characters when reading the story” and “I am not good at imagining what other people would feel when encountering an event.” High scores on these items revealed difficulties male students had in building good relationships in club activities and classrooms. According to previous studies, not only are relationships between peers very important for males, but making good friendships in class and club activities has a positive effect on mental health, while lack of friendships may have a negative effect on their SOC [41]. Males’ communication tends to be more direct than females’ [48] and problems may not surface while boys are in junior high school, but as high school students, when the relationship with close friends becomes more important, if males fail to communicate with their peers, they may lose their sense of belonging at school. Teachers need to pay attention to male students who are not good at communication, because the family is not always aware of what is going on at school. It may be helpful to give such boys specific advice on social skills at school.

### 4.6. The Relationship between ASD and SOC

In this study, we also examined the relationship between SOC and ASD and found a negative effect only on high school boys (Figure 1 and Figure 2). With regard to formation of SOC, it is important to have legitimate people who can be trusted [35]. However, ASD traits make it difficult to foster good and intimate relationships with others. Persons with ASD traits have difficulty understanding others’ intentions and having friendly communication [49]. In addition, people with a high SOC exhibit cognitive coping that easily adapts to their environment, such as changing goals according to the situation [50]. However, people with developmental disabilities tend to be more obsessed with ideas. Singling out and focusing on only unpleasant parts of events, having obsessions or rigidity of thought, and viewing choices as completely right or completely wrong are examples of autistic tendencies [51]. These characteristics can make it difficult to nurture a strong SOC. Depending on the communication characteristics of people with ASD, they may cause embarrassment to their friends, leading to mutual negative emotions. Being entangled in these negative emotions leads to increased stress and can be a cause of depression. It is extremely important for schoolteachers to pay attention to student communication and identify student characteristics to provide appropriate support. At the same time, it is necessary to accumulate and clarify future research as to why there was a significant association only with school boys.

This study has several limitations. First, parents’ response rate was low, while high school students had a high response rate, which may indicate that only parents who understood their children responded. Second, only mothers and not fathers were asked to participate. In the future, it will be necessary to invite both parents to participate. Finally, our study only examined students and mothers from one high school. In order to generalize findings, it will be necessary to conduct research in diverse schools representing people from various backgrounds.

## 5. Conclusions

Through our study, we gained knowledge about the relationship between mother and child SOC, which has received little attention to date. Female students’ SOC was slightly related to their mothers’, but male students’ SOC was not. This may be due to psychological closeness between daughters and mothers. The mothers’ SOC affected family conflicts as perceived by the daughters, which may in turn have impacted the daughters’ SOC. However, when children reach high school, their worlds expand; outside influences, such as school, become stronger than parental influences. Both male and female students’ SOC is positively impacted if they feel they are recognized by their schoolteachers. Lack of imagination in males had a negative impact on their SOC. Schools are the best place for providing intervention and support for students to develop a healthy SOC. Being sensitive to gender differences, providing information to families, and encouraging positive relationships with teachers at school may improve students’ SOC.

## Figures and Tables

**Table 1:** Participants’ characteristics.

	N	(%)/SD
**Mothers**	203	
Age (mean)	47.4	4.4
Employment		
Regular employment	49	(24.0)
Non-regular (part-time job)	122	(60.0)
Unemployed	29	(14.3)
Marital status		
Divorced/Bereaved	24	(11.8)
Married	177	(87.2)
Economic conditions		
Poor/very poor	87	(42.9)
Average	67	(33.0)
Wealthy/very wealthy	48	(23.6)
SOC ^1^, mean (SD), range: 13–65	42.7	7.6
**Students (*n* = 629)** ^2^		
Male	300	(47.7)
Female	329	(52.3)
1st grade	225	(35.8)
2nd grade	204	(32.4)
3rd grade	200	(31.8)
SOC, mean (SD), range: 13–65	37.3	6.8
**Students (*n* = 203)** ^2^		
Male	95	(46.7)
Female	108	(53.2)
1st grade	75	(37.0)
2nd grade	63	(31.0)
3rd grade	65	(32.0)
SOC, mean (SD), range: 13–65	36.9	6.9

^1^ SOC = Sense of coherence. Missing values are excluded from the table. ^2^ Repost.

**Table 2:** Mothers’ and students’ average total SOC ^1^ scores.

	SOC Score (Range 13–65)	SD	*p*-Value
**Mothers**			
Male children (*n* = 95)	42.8	6.8	0.238 ^2^
Female children (*n* = 108)	42.3	8.1	
**Students (Children, Matched with Parents)**			
Male (*n* = 95)	38.5	6.7	0.004 ^2^
Female (*n* = 108)	35.7	6.9	

^1^ SOC = Sense of coherence. *t*-test was performed between males and females. ^2^ *t*-test.

**Table 3:** Mother–child SOC correlation (mother–child common variables).

Mothers’ Variables		Male Students (*n* = 95)								Female Students (*n* = 108)							
		SOC ^1^ Total Score		FRI ^2^						SOC ^1^ Total Score		FRI ^2^					
				Cohesion		Expressiveness		Conflict				Cohesion		Expressiveness		Conflict	
		*r*	*p*	*r*	*p*	*r*	*p*	*r*	*p*	*r*	*p*	*r*	*p*	*r*	*p*	*r*	*p*
Mothers’ SOC score		0.068	0.569	-	-	-	-	-	-	0.226	0.023	-	-	-	-	-	-
Mothers’ FRI score																	
	Cohesion	0.193	0.085	0.392	<0.001	-	-	-		0.131	0.164	0.349	<0.001	-	-	-	-
	Expressiveness	0.028	0.806	-	-	0.196	0.085	-	-	0.174	0.063	-	-	0.290	0.002	-	-
	Conflict	−0.111	0.328	-	-	-	-	0.439	0.000	−0.200	0.033	-	-	-	-	0.561	<0.001

^1^ SOC = Sense of coherence. ^2^ FRI = Family Relationships Index.

**Table 4:** Correlation between students’ SOC ^1^ (by gender) and other variables.

Variables	Male Students’ Total SOC Score (*n* = 95)		Female Students’ Total SOC Score (*n* = 108)	
	*r*	*p*	*r*	*p*
Students’ FRI ^2^				
Cohesion	0.446	<0.001	0.243	0.009
Expressiveness	0.388	<0.001	0.436	<0.001
Conflict	−0.316	0.005	−0.325	<0.001
School membership scale				
Accepted by students	0.631	<0.001	0.468	<0.001
Accepted by teachers	0.648	<0.001	0.529	<0.001
Sense of belonging	0.667	<0.001	0.414	<0.001
Personality traits (AQ-J-16)				
Low communication ability	−0.400	<0.001	−0.345	<0.001
Low social skills	−0.217	0.052	−0.152	0.107
Lack of imagination	−0.339	0.012	−0.101	0.284
Difficulty in switching attention	−0.393	<0.001	−0.428	<0.001

^1^ SOC = Sense of coherence. ^2^ FRI = Family Relationships Index.

## Data Availability

The data in this study is a survey of parents and children at a high school in Tokyo and is very sensitive, including information on autism in relation to legal minors. In the surveyed school, disclosure of raw data is not allowed even if anonymous.
